# Quality appraisal of clinical practice guidelines addressing massage interventions using the AGREE II instrument

**DOI:** 10.1186/s13643-024-02503-6

**Published:** 2024-03-08

**Authors:** Mingyue Fan, Aolin Liu, Taoying Lu, Xiaowen Zhou, Chen Tian, Bingqing Liu, Qianwen Xie, Jianxiong Cai, Lingjia Yin, Long Ge, Darong Wu

**Affiliations:** 1 The Second Clinical Medical College, Guangzhou University of Chinese Medicine, Guangzhou, People’s Republic of China; 2 State Key Laboratory of Dampness Syndrome of Chinese Medicine, The Second Affiliated Hospital of Guangzhou University of Chinese Medicine, Guangzhou, People’s Republic of China; 3 Outcome assessment research team in Chinese medicine, Guangdong Provincial Hospital of Chinese Medicine, Guangzhou, People’s Republic of China; 4 Department of Social Science and Health Management, School of Public Health, Lanzhou University, Lanzhou, People’s Republic of China; 5 Karolinska Institutet, Stockholm, Sweden; 6 Key Laboratory of Evidence-Based Medicine and Knowledge Translation of Gansu Province, Lanzhou, People’s Republic of China; 7 Guangdong Provincial Key Laboratory of Clinical Research on Traditional Chinese Medicine Syndrome, The Second Affiliated Hospital of Guangzhou University of Chinese Medicine, Guangzhou, People’s Republic of China

**Keywords:** Massage, Practice guideline, Consensus, Recommendation, Quality assessment, AGREE II

## Abstract

**Objective:**

The purpose of this study was to systematically evaluate the methodological quality of massage-related clinical practice guidelines (CPGs)/consensus on massage using the Appraisal of Guidelines Research and Evaluation (AGREE II) instrument and to summarize the current status of recommendations in the CPGs.

**Methods:**

The Chinese National Knowledge Infrastructure (CNKI), WanFang Data, China Science and Technology Journal Database (VIP), China Biology Medicine disc (CBM), PubMed, Embase, and guideline websites (such as the Chinese Medical Ace Base, the China Association of Chinese Medicine, the World Health Organization, Guideline International Network, National Institute for Health and Care Excellence, Scottish Intercollegiate Guidelines Network) were searched from inception to October 31, 2022. In addition, the reference lists of relevant studies were reviewed to identify domestic and overseas massage CPGs/consensus. The search terms adopted a combination of subject words and free words, mainly including traditional Chinese medicine, complementary therapies, Tuina, massage, manipulation, chiropractic/osteopathic, spinal, acupressure, guideline, and consensus. Two researchers independently completed the eligible records and extracted the data. Before the formal research, calibrations were performed twice on AGREE II, and all reviewers completed the pilot test three times until they understood and reached an agreement on the assessment items. Three researchers appraised the methodological quality of the included guidelines using the AGREE II instrument and calculated the overall intraclass correlation coefficient (ICC) of agreement.

**Results:**

The evaluation results showed that among the 49 eligible CPGs/consensus, 4 (8.2%) CPGs/consensus were considered “recommended”, 15 (30.6%) CPGs/consensus were considered “recommended with modifications”, and 30 (61.2%) CPGs/consensus were considered “not recommended”, while the consensus was considered “not recommended”. Generally, the scores in the six domains of the guidelines were all higher than the consensus. Evaluation results for the overall quality of 36 CPGs showed that 4 (11%) were “good quality”, 15 (42%) were “sufficient quality” and 17 (47%) were “lower quality”. The AGREE II quality scores of domains ranged from 0.30 to 0.75 ([ICC = 0.993, 95% CI (0.992, 0.995)]). The domain of scope and purpose (domain 1), with a median score of 0.75 (0.52~0.91), performed best in the guidelines with AGREE II, and stakeholder involvement (domain 2) [median 0.39 (0.31~0.56)] and application (domain 5) [median 0.30 (0.17~0.47] obtained lower scores. The consensus score of domain 1 was better at 26.0 (21.6~44.8), followed by rigor of development (domain 3) with a score of 18.0 (10.0~28.9). A total of 119 massage-related recommendations were extracted from 49 guidelines/consensuses, including “in favor” (102, 85.7%), “against” (9, 7.6%), and “did not make recommendations” (8, 6.7%).

**Conclusion:**

The overall quality of the included guidelines was low, and most of the guidelines were not “recommended”. In future guideline updates, the existing evidence should be used, the professional composition of members of the expert group should be enriched, and patients’ values and preferences should be fully considered. It is necessary to clearly propose recognizable recommendations and strengthen the rigor and standardization of guideline formulation. Thus, clear standard guidelines can be formulated to better guide clinical practice.

**Supplementary Information:**

The online version contains supplementary material available at 10.1186/s13643-024-02503-6.

## Introduction

Massage dates back to at least the second century B.C. and is generally defined as the manipulation of soft tissue [1, 2]. It is one of the oldest therapeutic techniques to which people around the world attach importance [3]. It can improve microcirculation and regulate the human body’s subhealth and health conditions by manipulating muscles or connective tissues [4].

Massage therapy is a widespread and beneficial intervention of complementary medicine and has been well recognized and adopted in clinical practice. Some reviews have summarized the clinical applications of massage therapy for various diseases, including improving health and development in preterm/low-birth weight infants, reducing pain and anxiety, and treating some respiratory and digestive system diseases [5, 6]. In a systematic review of abdominal massage, massage was used for adult digestive disorders, pediatric disorders, gynecological disorders, obstetric disorders, metabolic disorders, and psychological disorders [7]. In the absence of sufficiently effective and safe pharmacological treatments for these diseases, massage as a nonpharmacological therapy has become a viable means of treating these diseases by avoiding possible side effects while reducing pain and discomfort [8].

With the widespread use of massage therapy, clinical evidence of the use of massage for various diseases or symptoms is rapidly growing. A recent review summarized the evidence related to pediatric massage based on 38 published systematic and nonsystematic reviews. The results presented more positive effects than lack of effect, and no negative effects were found in four major outcome groups with regard to physical and metabolic aspects, well-being and quality of life, mental health and behavior, and management [9]. For the improvement of psychological variables and subjective symptoms, such as pain and quality of life, there appears to be better evidence [10], which provides an important basis for the clinical application of massage.

With the development of complementary and alternative medicine, massage therapy is no longer supported solely by the personal experience of the physician or practitioner but is also supported by high-quality scientific evidence. Some clinical practice guidelines (CPGs) on massage have been developed to assist physicians and practitioners with the integration of evidence into clinical decision-making. These CPGs involve acute or chronic pain [11], cancer [12], and some digestive system diseases of children [13, 14].

High-quality CPGs are a decision-making tool to narrow the gap between current best evidence and clinical practice. They can help healthcare providers balance the risks and benefits of therapies, which ultimately leads to better patient outcomes and improves medical quality [15, 16]. Therefore, it is very important to assess the quality of CPGs.

Although many massage-related CPGs/consensuses have been developed internationally and have played a positive role in promoting the standardized use and treatment of disease with massage therapy, the quality of these guidelines/consensuses is not clear. Therefore, the integration of guidance evidence is necessary. This study systematically evaluated the methodological quality of massage-related CPGs/consensus using the Appraisal of Guidelines Research & Evaluation (AGREE II) instrument (available at http://www.agreetrust.org/) and summarized the current status of recommendations in the CPGs.

## Methods

### Eligibility criteria

No restriction was placed on classifications of massage, and eligible CPGs/consensus were included with reference to the “PICAR” framework [17].

1. Population, interventions, and comparators

The population of eligible CPGs/consensus for patients who require massage intervention. This review does not state ‘comparators’.

(2) Attributes of the CPG/consensus

The following criteria were used: (1) the title or abstract included the keywords ‘CPG’ or ‘guideline’ or ‘guidelines’ or ‘guidance’ or ‘consensus’; (2) the full text included ‘massage’ or ‘chiropractic’ or ‘acupressure’ or ‘manipulation’ or ‘osteopathic’ or ‘Tuina’ or ‘spinal’; (3) CPGs/consensus included recommendations related to ‘massage’ or ‘chiropractic’ or ‘acupressure’ or ‘manipulation’ or ‘osteopathic’; (4) CPGs/consensus were released or published in scientific paper or were the latest versions of CPGs available when multiple versions exist.

We excluded CPGs according to the following criteria: (1) full text of guidelines was not available; (2) earlier versions of guidelines with an available updated version, secondary or multiple publications; (3) interpretation or translation of guidelines/consensus, abstract of submission, systematic reviews, narrative reviews, primary studies, critical/clinical pathways, training manuals for medical professionals, textbook-like publications, guidelines for patients, editorials, translations of foreign guidelines or short summaries; (4) did not contain recommendations on ‘massage’ or ‘chiropractic’ or ‘acupressure’ or ‘manipulation’ or ‘osteopathic’; and (5) guidelines published in languages other than Chinese or English.

### Search strategy

The literature search was conducted by two reviewers (M.Y.F., B.Q.L.) from inception to October 31, 2022. The search was limited to humans and the Chinese or English language. The systematic literature search was conducted in the following databases: Chinese Biomedical Literature database (http://www.sinomed.ac.cn/), WanFang database (Chinese Medicine Premier, http://www.wanfangdata.com.cn/), VIP (Chinese journals full-text database, http://data.whlib.ac.cn/), China National Knowledge Infrastructure (http://www.cnki.net/), China Biology Medicine disc (www.sinomed.ac.cn), PubMed (https://pubmed.ncbi.nlm.nih.gov), Excerpta Medica Database (https://www.embase.com), and guideline websites, such as the Chinese Medical Ace Base (http://seleguide.yiigle.com/), the China Association of Chinese Medicine (http://www.cacm.org.cn/category/), the World Health Organization (https://www.who.int), Guideline International Network (https://guidelines.ebmportal.com), National Institute for Health and Care Excellence (https://www.nice.org.uk), Canadian Medical Association CPG Infobase (https://joulecma.ca/cpg/), Scottish Intercollegiate Guidelines Network(https://www.sign.ac.uk/). Search terms were (“guidelines as topic” OR “guideline” OR guideline* OR guidance OR recommendation* OR consensus) AND (massage OR chiropractic OR acupressure OR “massage” OR “chiropractic” OR “acupressure” OR “Tuina” OR “manipulation” OR “osteopathic” OR “spinal”) AND (“Complementary Therapies” OR “Medicine, East Asian Traditional” OR complementary OR “East Asian Traditional” OR TCM OR “Chinese medicine” OR “traditional Chinese” OR “traditional medicine” OR “alternative” OR “oriental medicine” OR “east Asian medicine”) AND “human” OR human. Detailed construction of these search strategies is attached in Supplementary Material: Appendix 1.

### CPGs/consensus selection and data extraction

Two reviewers (M.Y.F. and B.Q.L.) independently imported the bibliography into EndNote X9 and removed duplicates from the bibliography. Then, Microsoft Excel 2021 was used to screen the titles and abstracts. Finally, we screened the full texts to identify the included CPGs/consensus. Disagreements were resolved by consensus or a third reviewer (C.T.).

Two reviewers (M.Y.F. and X.W.Z.) independently extracted descriptive information from the included CPGs/consensus and cross-checked it to ensure data quality. The following three sections were extracted from the included CPGs/consensus: general characteristics of CPGs/consensus (title, authorship list, date of publication, organization/society that developed the guidelines, target users, sponsoring organization, country, updated/original, target population, disease classification, age group of target population, definition of massage/Tuina, search year covered, methods used to determine recommendations); characteristics of guidelines concerning the contents of rigor of development (systematic search, databases, comprehensive search strategies, study basis for massage recommendations, methods used to determine recommendations, peer review); general recommendations (criteria for rating evidence, criteria for grading recommendation, disease classification, number of recommendations related to massage/Tuina, population, alone or with other interventions, direction of the recommendation, basis for recommendation, certainty of evidence, strength of recommendation, type of intervention).

### Assessment for guideline quality and investigation of heterogeneity

The Appraisal of Guidelines Research & Evaluation (AGREE II) instrument [18–20] is a tool used to assess the methodological quality of CPGs. It was translated into Chinese by Li Min Xie and Wenyue Wang at Guang’anmen Hospital [21]. The AGREE II scale is composed of 23 items grouped into 6 domains using a seven-point scale from 1 for “strongly disagree” to 7 for “strongly agree”. Based on examples and instructions in the AGREE II manual [18], the appraisers rated each of the AGREE II items and the two global rating items.

The total was calculated by summing the scores of all items within the domain and scaling the total as a percentage of the maximum possible score for that domain. A scaled domain percentage score was calculated according to the AGREE II methodology as follows:

$$\frac{\textrm{obtained}\ \textrm{score}-\textrm{minimum}\ \textrm{possible}\ \textrm{score}}{\textrm{maximum}\ \textrm{possible}\ \textrm{score}-\textrm{minimum}\ \textrm{possible}\ \textrm{score}}$$

To reflect the recommendation intention after the overall quality assessment of each CPG/consensus, the overall score was obtained by calculating the sum of six domain scores and dividing by 600%. The total score range was 0–100% [22, 23]. The domain scores were categorized as good quality (≥ 70%), sufficient quality (50–70%), and lower quality (< 50%), which indicated corresponding recommendation intentions for each CPG as “recommended” (≥ 70%), “recommended with modifications” (50–70%) or “not recommended” (< 50%). The ‘obtained score’ was the sum of the appraisers’ scores for each item, making it possible to consider the natural discrepancies between the two appraisers.

Three appraisers (M.Y.F., X.W.Z., and C.T.), including a guideline methodologist, received similar training in regard to the process and methods of guideline development as well as the application of the AGREE II instrument. They were pilot-tested before they independently conducted the CPG evaluation. Except for the guideline methodologist, the other two appraisers were clinicians with experience in massage and health care improvement. The overall evaluations, including recommend, recommend with modifications or do not recommend, were independently determined by each appraiser. Every guideline was assessed by at least two assessors.

### Statistical analysis

The characteristics of the included CPGs and consensus are depicted as the number of guidelines and the proportion to the total number of guidelines (*N* (%)). For the AGREE II quality assessment, the scores of all eligible guidelines from three appraisers were summarized and calculated and were presented as the median and 25–75% (*M* (*P*_*25*_ *~P*_*75*_)) and mean and standard deviation (SD) values, which showed the proportion of standardized scores for each domain of the guideline and the consensus. The agreement among appraisers was calculated using the intraclass correlation coefficient (ICC) [24], defined as follows: < 0.20, poor; 0.21–0.40, fair; 0.41–0.60, moderate; 0.61–0.80, good, 0.81–1.00, very good [25, 26]. ICC calculations were performed using the Statistical Package for Social Science (SPSS) software (version 18).

## Results

### Literature search and selection

**Fig. 1:**
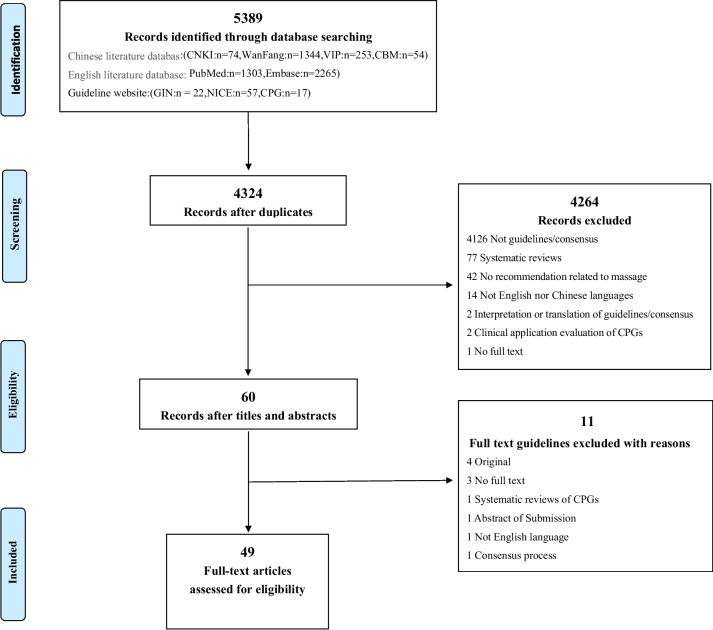
Flow chart of the selection process

The searches retrieved 5389 hits, of which, we excluded 1065 duplicates and 4264 records after screening titles and abstracts, leaving 60 full-text articles that were screened and 49 full-text articles were assessed for eligibility. Of the 60 full texts, we excluded 11 articles for the following reasons: 1 was a systematic review of CPGs related to massage, 1 was not in English nor Chinese languages, 1 was an abstract, 1 was by consensus process, 4 were original versions, and 3 were not available as full text. The process for selecting the articles is presented in Fig. 1. The ggplot2 package of R studio (v 2022.12.0-353) was used for raincloud plotting, and the bubble plot depicting the assessment results of guidelines and CPGs concerning different disease types was processed in Bioinformatics (https://www.bioinformatics.com.cn/) [27].

### Characteristics of included CPGs and consensus

Forty-nine articles were included; 36 (73.5%) of them were CPGs and 13 (26.5%) were expert consensus. The included guidelines/consensus were mainly developed from organizations or societies, a majority of which were located in America (25, 51.0%). The CPGs were published between 2006 and 2022, and 20 CPGs (11.54%) were updated versions. Among these, 18 (36.7%) guidelines used Grading of Recommendations Assessment, Development, and Evaluation (GRADE) to assess the certainty of the evidence, and the other 18 (36.7%) used GRADE to assess the strength of the recommendation. The eligible CPGs and consensus characteristics are illustrated in Table 1 and Supplementary Material Appendix 2.

**Table 1:** General characteristics of included CPGs and consensus

Variable	*N* (%)
Type of literature	
CPG	36 (73.5)
Expert consensus	13 (26.5)
Region of organization/society responsible for CPGs/consensus development	
America	25 (51.0)
Asia	16 (32.7)
Europe	7 (14.3)
Oceania	1 (2.0)
Original/updated	
Original	29 (59.2)
Updated	20 (40.8)
Recommendation intention according to overall quality assessment of CPGs/consensus	
Recommended	4 (8.2)
Recommended with modifications	15 (30.6)
Not recommend	30 (61.2)

### AGREE II quality scores

The ICC analysis showed very good agreement among the three reviewers [ICC = 0.993, 95% CI (0.992, 0.995)].

**Fig. 2:**
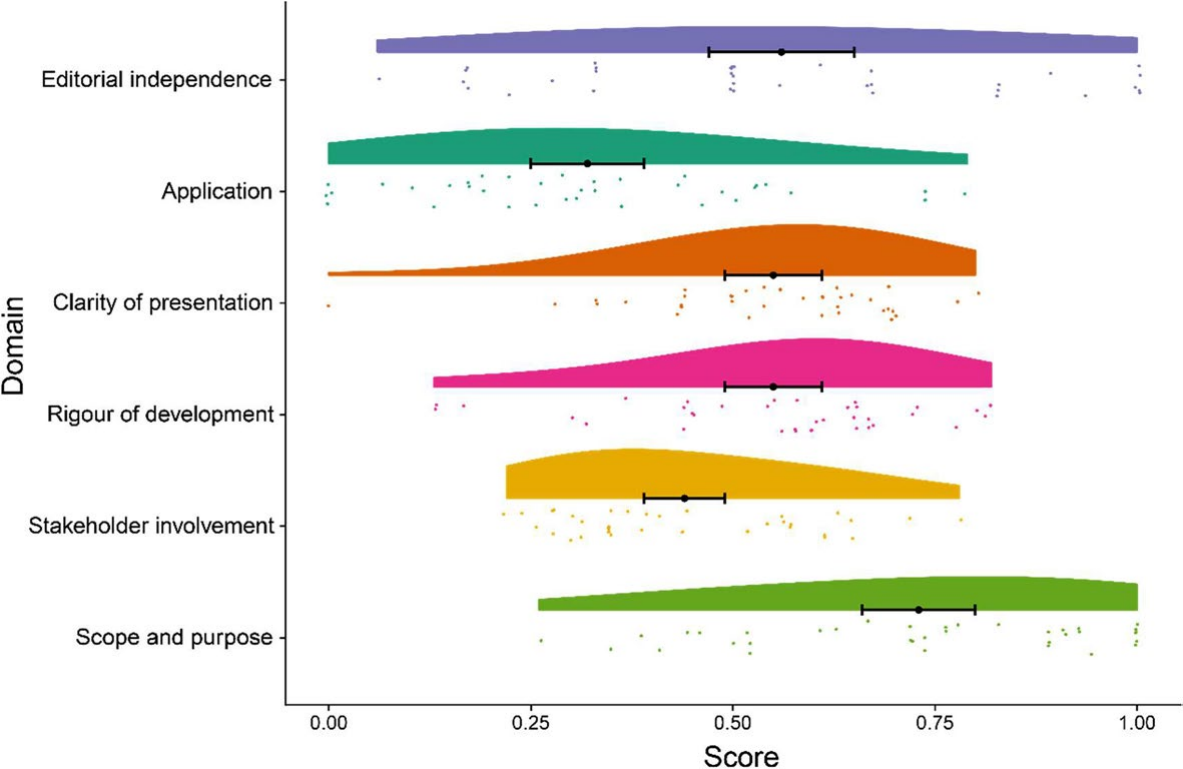
AGREE II assessment by domain of 36 guidelines. The raincloud plot with Mean score ± 95% CI comprehensively depicts the distribution of the AGREE II score of the guidelines in each domain. Each dot exhibits the standard value combined assessment of three researchers concerning each guideline

Evaluation results for the overall quality of 36 CPGs showed that 4 (11%) were “good quality”, 15 (42%) were “sufficient quality” and 17 (47%) were “lower quality”. The AGREE II quality scores of domains ranged from 0.30 to 0.75 (see Fig. 2). The domain with the highest score across the guidelines was scope and purpose, with a median of 0.75 (0.52~0.91). The stakeholder involvement domain [median: 0.39 (0.31~0.56)] and application domain [median: 0.30 (0.17~0.47] obtained lower scores. Each domain presented different results in various guidelines (see Table 2). AGREE II scores of each eligible CPG/consensus are presented in Supplementary Material Appendix 3.

**Table 2:** AGREE II assessment scores of six domains of eligible CPGs

Domains	Content	Median score (IQR, %)	Mean score (X±SD, %)	Score segmentation (number of included guidelines, %)			
				< 25%	25%~50%	50%~75%	> 75%
1	Scope and purpose	75.0 (52.0~92.5)	73.1±21.9	0 (0.0)	6 (16.7)	12 (33.3)	18 (50.0)
2	Stakeholder involvement	39.0 (31.0~56.8)	43.7±15.0	2 (5.6)	21 (58.3)	12 (33.3)	1 (2.8)
3	Rigor of development	59.0 (44.3~66.5)	55.1±18.0	3 (8.3)	9 (25.0)	20 (55.6)	4 (11.1)
4	Clarity of presentation	57.5 (44.0~66.5)	54.7±15.9	1 (2.8)	9 (25.0)	24 (66.7)	2 (5.6)
5	Applicability	30.0 (17.0~48.3)	31.9±21.2	14 (38.9)	14 (38.9)	7 (19.4)	1 (2.8)
6	Editorial independence	50.0 (33.0~83.0)	56.1±28.2	6 (16.7)	5 (13.9)	15 (41.7)	10 (27.8)

#### Scope and purpose

The average score of the six included guidelines in terms of the scope and purpose domain was 0.73, 95% CI (52.0~92.5), ranging from 0.26 to 1.00 [28–33]. Most eligible guidelines comprehensively described the overall purpose, health questions and target populations, except for 5 guidelines [13, 34–37] that did not describe the health intents, expected benefit/outcome, or target population, three guidelines [36–38] that did not provide a detailed description of PICO questions, i.e., population, intervention or exposure, comparative and study outcomes, and 1 guideline [39] that did not explicitly describe the details of the target population.

#### Stakeholder involvement

The overall score in this domain was low; the average score was 0.44, 95% CI (31.0~56.8). All CPGs reported comprehensive member information of the guideline development group. Ten CPGs [11, 12, 33, 34, 36, 38, 40–43] did not mention the patient’s views and preferences, while the target users were not clearly defined in nine CPGs [34, 36, 39–41, 44–47].

#### Rigor of development

The mean score for this domain was 0.55, 95% CI (44.3~66.5). Twenty-four guidelines scored above 50%, and three guidelines scored below 25%. Five guidelines [35–38, 48] did not provide detailed search strategies. The inclusion/exclusion criteria were explicitly described in eight guidelines [12, 32, 40, 41, 49–52]. Most guidelines clearly described the strengths or limitations of the body of evidence and health benefits, harms or risks of side effects. Four CPGs [37–39, 48] did not report criteria for rating evidence, and seven CPGs [11, 34, 37, 41, 48, 49, 53] did not address the methods for formulating the recommendations. Two CPGs [38, 42] did not mention benefits, harms or the balance between them. Only 1 guideline [31] provided comprehensive updated information.

#### Clarity of presentation

In the clarity of presentation domain, the mean score was 0.55, 95% CI (44.0~66.5). We found that two CPGs [32, 42] lacked specific and unambiguous recommendations. In five CPGs [13, 14, 34, 42, 54], multiple options with detailed population or clinical situation descriptions were provided for each targeted question, and key recommendations were presented in unclear ways, i.e., they could not be clearly recognized in the texts of those CPGs [32, 42, 49].

#### Applicability

The score for the application domain was 31.9% ± 21.2%, 95% CI (17.0~48.3). The potential resource implications, details of the described facilitators, or barriers to application were not clearly defined in most CPGs, except for six CPGs [12, 13, 32, 33, 36, 47] that identified the types of facilitators and barriers. Facilitators included a wide variety of locations for therapy implementation [12, 13], supportive policy from the local government, standardized training procedures provided to the practitioners [13], etc. Several barriers mentioned in those CPGs which might impact the guideline implementation, such as lack of availability in community hospitals [33], loss of skill over time from disuse, inadequate office space [32], etc.

Four guidelines [14, 28, 33, 55] mentioned information regarding the facilitators and barriers to implementing recommendations, and five guidelines [31, 32, 39, 55, 56] provided advice and tools on how the recommendations could be put into practice. In addition, only three guidelines [56–58] fully considered the potential resource implications of applying the recommendations, and two guidelines [51, 57] completely presented performance monitoring indicators and auditing criteria, including advice on the frequency and interval of measurement descriptions and operational definitions of how the criteria should be measured.

#### Editorial independence

This domain obtained a mean score of 56.1% ± 28.2%, 95% CI (33.0~83.0). Four CPGs [12, 45, 46, 59] did not state that the views of the funding body had not influenced the content of the guidelines, 4 CPGs [11, 35, 41, 49] did not present the conflicts of interest of the guideline development group members, while 1 CPG failed to declare both [37].

The overall assessment ratings for the 13 consensuses evaluated ranged from 0.06 to 0.46. All consensus were classified as “lower quality”. For 13 consensuses the average scores of AGREE II domains 1–6 were 33.2%, 18.0%,19.4%, 9.83%, 18.0% and 26.9%, respectively (see Table 3). It shows that each domain needs to be improved. The consensus lacks recommendations, which leads to a low rating in the ‘Clarity of presentation’ domain. Comparing with consensus, the development of CPGs is more rigorous, structured and reliably organized.

**Table 3:** AGREE II evaluation results of guidelines and consensuses

Domains	Content	Guidelines (mean,%)	Consensuses (mean,%)
1	Scope and purpose	73.1%	33.2%
2	Stakeholder involvement	43.7%	18.0%
3	Rigor of development	55.1%	19.4%
4	Clarity of presentation	54.7%	9.8%
5	Applicability	31.9%	18.0%
6	Editorial independence	56.1%	26.9%

number of guidelines was 36; number of consensus was 13

### Level of evidence and strength of recommendation

Thirty-four CPGs (83.33%) used 10 types of grading systems to rate the level of evidence and the strength of recommendation (see Table 4). The GRADE system with wider acceptance was adopted in the development of 16 CPGs. The grading system of evidence and recommendation was not reported in 2 guidelines [39, 48]

**Table 4:** Grading system of evidence and strength of recommendation

Grading system	Criteria for rating evidence	Criteria for grading recommendation (*N*)	Number of guidelines	Organization/author responsible for guideline development
GRADE	A, B, C, D	1 (2), 2 (10)	16	CCGI, ACP, AOA, ACCP, TCM Recs, ASCO, JOA, NICE, John W. Devlin, SD Guy,
System developed by OPTIMa Collaboration	/	Offer, consider (5), do not offer (3)	2	OPTIMa
SIGN	1++, 1+, 1-, 2++, 2+, 2-, 3, 4	A, B, C, D	4	BGS, CCGPP, NICE
System recommended by The Cochrane Collaboration Back Review Group and Oxman and Guyatt	A, B, C, D	Recommended (4), Consider using (1), we cannot recommend (3), we do not recommend	2	COST B13, CCA
ACCP	High, moderate, low	Strong, weak (1)	1	ACCP
USPSTF	High, Moderate, Low	A, B (2), C (1), D, I (2)	1	SIO
Combined Grading system	A, B, C+, C, D, D+, D-	Strongly recommended (12), suggested use (4), neutral, suggested no use, strongly not recommended	2	OP
Ranking of the Canadian Task Force on Preventive Health Care	I, II-1, II-2, II-3, III	A, B (1), C, D, E, I	1	SOGC
Classification criteria of TCM literature	I, II, III, IV, V	A, B, C, D, E	1	Chen
CPRC	I, II, III, IV	A, B (7), C, D (2)		CPRC

*N* number of recommendations of corresponding grade. Combined grading system: This grading system was developed according to the Ottawa Panel for alphabetical grading system and to the Cochrane Collaboration for international nominal grading system

*GRADE* Grading of Recommendations Assessment, Development and Evaluation, *SIGN* Scottish Intercollegiate Guidelines Network, *OPTIMa* Ontario Protocol for Traffic Injury Management Collaboration, *ACCP* American College of Chest Physicians, *USPSTF* U.S. Preventive Services Task Force, *COST B13* COST B13 Working Group on Guidelines for Chronic Low Back Pain, *SIO* the Society for Integrative Oncology, *BGS* the British Geriatrics Society, *CCA* Canadian Chiropractic Association, *CCGI* The Canadian Chiropractic Guideline Initiative, *ACP* The American College of Physicians, *CCGPP* Council on Chiropractic Guidelines and Practice Parameters, *AOA* The American Osteopathic Association, *TCM Recs* Trustworthy Traditional Chinese Medicine Recommendations Working Group, *COTB* Orthopedics and Traumatology Branch of China Association of Chinese Medicine (CACM), *AIMSS* Australian Institute for Musculoskeletal Science, *ASCO* American Society of Clinical Oncology, *JOA* Japanese Orthopaedic Association, *NICE* the National Institute for Health and Clinical Excellence, *SOGC* the Society of Obstetricians and Gynaecologists of Canada Clinical Practice-Gynaecology, *CSMC* Professional Committee of Spine Medicine of Chinese Association of integrated Traditional and western medicine (CAIM), *CPRC* Pediatric Rehabilitation Committee of China Association Rehabilitation Medicine (CARM)

### Recommendations for massage interventions

We included 11 massage-specific CPGs/ consensuses and 38 disease-based CPGs/ consensuses with recommendations in terms of massage.

### General view of the recommendations

A total of 119 massage-related recommendations were extracted from 36 guidelines. It included “in favor” (102, 85.7%), which meant the massage was recommended for use. For instance, in the CPGs applied GRADE [13, 14], in favor was divided into “strong” or “weak” levels; and the same for “against” (9, 7.6%), which meant the massage wasn’t recommended for use. It was also divided into “strong” or “weak” based on GRADE according to CPGs [13, 14]. For those addressed “did not make recommendations” (8, 6.7%), some of the CPGs provided certain circumstances under which massage was not recommended, e.g., spinal manipulation cannot be recommended for the management of patients with episodic tension-type headache [40], though others did not mention the contents.

### Target population

**Fig. 3:**
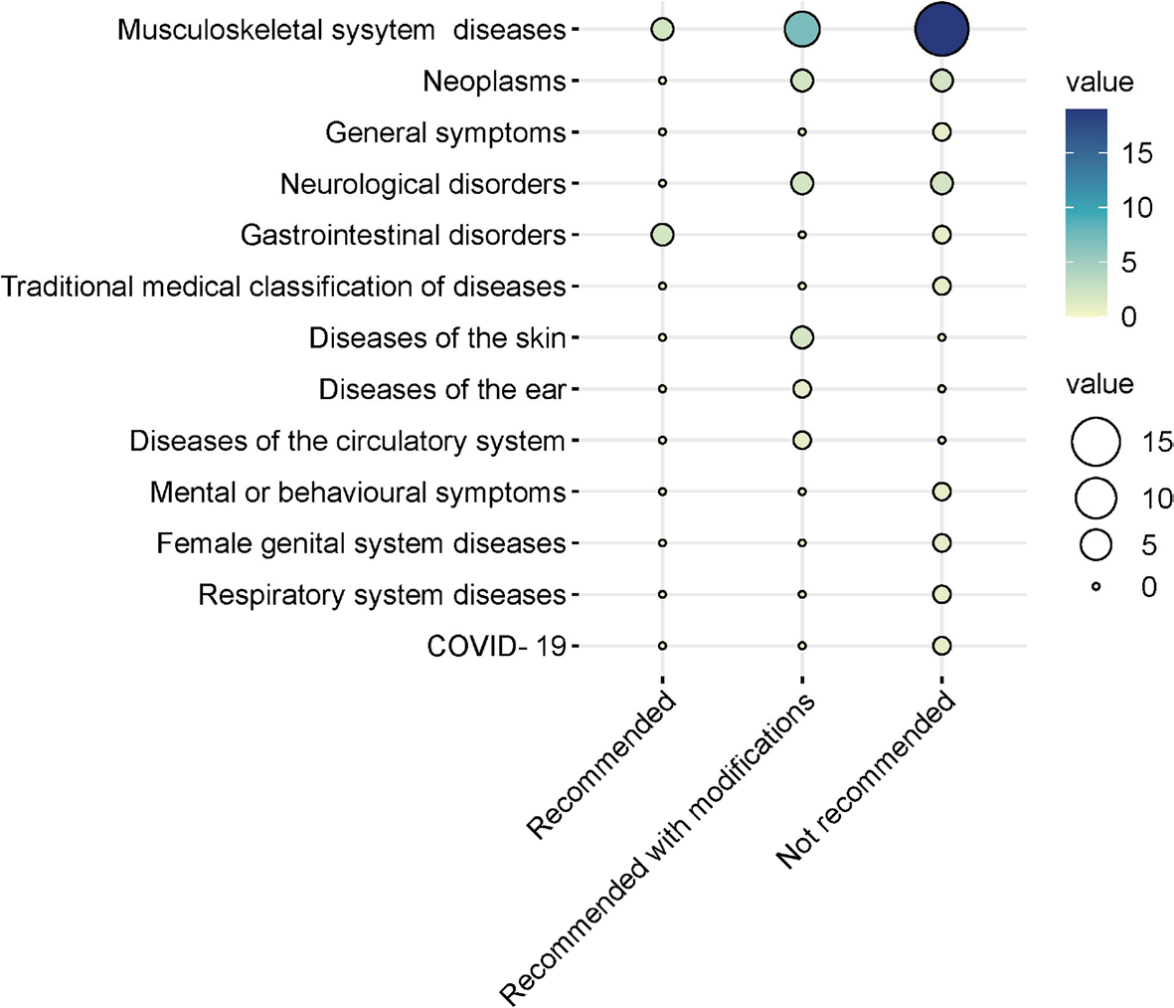
Different disease types covering massage recommendations and quality assessment of guidelines and consensus based on AGREE II. The size and color of the circle represent the number of recommendations, as the number increases, the circles become larger and darker

Figure 3 shows that the target populations of 9 (18.4%) CPGs and consensus were children and adolescents (< 18 years), while 24 (49%) targeted middle-aged adults and elderly people (≥ 18 years) and the general population (16, 32.7%). Massage-related diseases were classified according to the International Classification of Diseases 11th Revision (ICD-11). Most (28, 57.1%) were musculoskeletal system diseases. The evaluation results showed that among the 49 eligible CPGs/consensuses, 4 (8.2%) CPGs/consensuses were considered “recommended”, 15 (30.6%) CPGs/consensuses were considered “recommended with modifications”, and 30 (61.2%) CPGs/consensuses were considered “not recommended”.

### Intervention characteristics

Table 5 shows that massage intervention characteristics were divided into two types, including massage interventions alone, which accounted for a large proportion (84, 70.59%).

**Table 5:** Massage interventions characteristics

**Massage alone or with other interventions**	***N*** **(%)**
Alone	84 (70.59)
With conventional medicine	9 (7.56)
With Chinese herbal medicine	1 ( 0.84)
With non-pharmacological intentions	22 (18.49)
Other	3 (2.52)
Type of massage interventions	N (%)
Massage/Tuina	47 (39.50)
Acupressure	8 (6.72)
Manipulation	32 (26.89)
Structural massage	1 (0.84)
Relaxation massage	1 (0.84)
Reptrotherapy	1 (0.84)
Manual therapy	9 (7.56)
Compound manipulation with anesthesia	1 (0.84)
Therapeutic massage	2 (1.68)
Other	8 (6.72)
Not reported	9 (7.56)

## Discussion

### Summary of the findings

To the best of our knowledge, this article is the first systematic and comprehensive assessment of the quality of current CPGs available for massage. We assessed the methodological quality of 49 massage-related CPGs/consensuses and extracted 119 massage-related recommendations from the included CPGs/consensuses, among which the “in favor” recommendations accounted for a large proportion of total recommendations (102, 85.7%). Developed/updated guidelines tended to have higher quality than earlier versions. Evidence-based guidelines scored consistently higher in all domains. A lack of international authoritative instruments in the appraisal of consensus might result in insufficient authenticity of evaluation.

### Relation to other studies

Several previous studies have evaluated the quality of massage-related guidelines, but these studies only focused on certain forms of massage or certain diseases. For example, a previous study assessed four guidelines for spinal manipulation in a study of complementary and alternative medicine (CAM) guidelines. In overall recommendations, these four guidelines were rated “yes, with modifications” [60]. However, in our studies, more than half of the guidelines/consensus was assessed as “not recommended”. The difference in results was likely due to the focus of the CAM guidelines on spinal manipulation, which also explains their failure to provide the broad range of massage guidelines that our study describes. In another quality appraisal of CPGs regarding nonpharmacological interventions for breast cancer survivors [61], massage was rated as “recommended” to alleviate the symptoms of anxiety, depression and distress in breast cancer survivors. The level of recommendation was also inconsistent with our study, which was “not recommended”.

### Strengths and limitations

Our study identified several strengths. First, the study was the latest methodological quality study that evaluated the quality of CPGS addressing massage interventions using the AGREE II instrument. Second, our study consisted of experienced clinical experts and methodologists in CPGs, to guide the guideline evaluation process. Third, we performed a systematic search of the literature to ensure the reliability of the findings.

Nevertheless, our study also had some limitations. We only assessed guidelines published in commonly used databases, which may not represent all massage guidelines. Guidelines published in other forms (i.e., books, booklets, or government documents) may have been missed. We only included guidelines published in Chinese or English, and some non-Chinese or non-English guidelines might have been missed. Thus, we may have underestimated guideline quality in some instances.

### Proposals for improving the quality of massage guidelines and consensuses

From 49 included CPGs/consensuses, 28 CPGs/consensuses were musculoskeletal system diseases, of which 9 were rated “recommended” or “recommended with modifications” and 19 were “not recommended”. This indicated that the current evidence was inconsistent in supporting the efficacy of massage in treating musculoskeletal system diseases. Similarly, the quality of evidence was inconsistent or poor for some other diseases, which may lead to a lack of supporting evidence available for physicians or practitioners. Therefore, we suggest that massage CPGs/consensus developers focus on improving the quality of massage therapy recommendations.

The overall quality of guidelines related to massage was low. Development of guidelines must follow a rigorous set of procedures [62], e.g., target audience, systematic review, evidence retrieval and synthesis, formulate recommendations, convene meetings, implementation, publishing, and updating. Guideline developers should pay more attention to the clarity of presentation, rigor of development, and applicability according to our study. In addition to the low quality of the CPGs, lack of well recognized placebo/sham control or poor comparability of individualized practitioner-based therapy may directly affect the quality of the massage research.

In accordance with AGREE II, there were two domains showed low scores based on our research, potential reasons might be few CPGs considered patients’ values/preferences or the absence of target users’ clarification/definition for “stakeholder involvement” domain; in the “application” domain, seldom implementation recommendations or applicable instruments were provided, less considerations on massage related resource implications, and inadequate detailed suggestions of massage interventions, such as frequencies or episodes for each acupoints due to different age groups, could be obtained. The high rating in the ‘scope and purpose’ domain reflected that objective(s) of the guideline, Population/Intervention/Comparison/Outcome/Study design (PICOs), and target population were comprehensively described.

In future guideline updates, the existing evidence should be reasonably adopted, the professional composition of the members of the expert group should be enriched, and patients’ values and preferences should be fully considered. It is necessary to propose recognizable recommendations and strengthen the rigor and standardization of guideline formulation to formulate clear and standard guidelines to better guide clinical practice.

### Implications

The development of the CPGs involves health promotion, screening, therapy, diagnosis, or prognosis [63]. Improved quality of guidelines may benefit all stakeholders, including healthcare workers, patients, and healthy individuals [64]. The evidence-based massage CPGs provided unbiased recommendations that were effective, safe and appropriate for patients, helping avoid ineffective or potentially harmful options [63]. CPGs may transform healthcare delivery and enhance patient outcomes [65]. Therefore, efforts must be made to guarantee the improvement of the quality of the CPGs. Our study also provided an exemplary practical approach to the quality evaluation of other guidelines.

## Future research directions

The findings of this article also provided some future research directions. First, the quality of the guidelines was low. On one hand, we need to focus on improving the quality of the CPGs to guide clinical practice. On the other hand, we need to conduct well-powered randomized controlled trials to improve the evidence bases. Second, through real-world study, we can obtain data on the advantageous diseases treated with massage. It will be helpful for researchers or doctors to conduct clinical trials and evaluate its clinical efficacy. Third, we may also develop some massage-specialized CPGs for the treatment of advantageous diseases, which would be valuable complementation for disease-based guidelines adopted by non-pharmacological therapies.

### Supplementary Information

**Additional file 1: Appendix 1.** Detailed construction of CPGs/consensus search strategies.

**Additional file 2: Appendix 2.** Characteristics of guidelines concerning the contents of rigor of development.

**Additional file 3: Appendix 3.** The AGREE II scores of each eligible CPGs/consensus.

## Data Availability

The data that support the findings of this study are available from the corresponding author upon reasonable request.
