# Supplementary material for: Quality appraisal of clinical practice guidelines addressing massage interventions using the AGREE II instrument
Source: Syst Rev. 2024 Mar 8;13:83. doi: 10.1186/s13643-024-02503-6 (PMC10921609; doi:10.1186/s13643-024-02503-6)
Supplement: Supplementary file 1 — Additional file 1: Appendix 1. Detailed construction of CPGs/consensus search strategies. [file 13643_2024_2503_MOESM1_ESM.docx]

Detailed construction of CPGs/consensus search strategies

**CNKI**

(KY=推拿 OR TI=推拿 OR KY=按摩 OR TI=按摩 OR KY=手法 OR TI= 手法 OR KY=捏脊 OR TI=捏脊 OR KY=捏积 OR TI=捏积 OR KY=穴位按压 OR TI=穴位按压) AND (KY=指南 OR TI=指南 OR KY=实践指南 OR TI=实践指南 OR KY=临床指南 OR TI= 临床指南 OR KY=临床实践 OR TI=临床实践 OR KY=临床实践指南 OR TI=临床实践指南 OR KY=共识 OR TI=共识 OR KY=专家共识 OR TI=专家共识 OR KY=专业共识 OR TI=专业共识)

**WanFang**

(题名或关键词:（"推拿" or "按摩" or "手法" or "捏脊" or "捏积" or "穴位按压")) and (题名或关键词: （"指南" or "实践指南" or "临床指南" or "临床实践" or "临床实践指南" or "共识" or "专家共识" or "专业共识"))

**VIP**

M=(推拿 OR 按摩 OR 手法 OR 捏脊 OR 捏积 OR 穴位按压) AND M=(指南 OR 实践指南 OR 临床指南 OR 临床实践 OR 临床实践指南 OR 共识 OR 专家共识 OR 专业共识)

**CBM**

#1 ( "推拿"[加权:扩展] OR "按摩"[加权:扩展] OR " 手法"[加权:扩展] OR "捏脊"[加权:扩展] OR "捏积"[加权:扩展] OR " 穴位按压"[加权:扩展]) OR( "推拿"[关键词:智能] OR "按摩"[关键词:智能] OR " 手法"[关键词:智能] OR "捏脊"[关键词:智能] OR "捏积"[关键词:智能] OR " 穴位按压"[关键词:智能]) OR( "推拿"[中文标题:智能] OR "按摩"[中文标题:智能] OR " 手法"[中文标题:智能] OR "捏脊"[中文标题:智能] OR "捏积"[中文标题:智能] OR " 穴位按压"[中文标题:智能])

#2 ( "指南"[加权:扩展] OR "实践指南"[加权:扩展] OR " 临床指南"[加权:扩展] OR "临床实践"[加权:扩展] OR "临床实践指南"[加权:扩展] OR " 共识"[加权:扩展] OR "专家共识"[加权:扩展] OR "专业共识"[加权:扩展]) OR ( "指南"[关键词:智能] OR "实践指南"[关键词:智能] OR "临床指南"[关键词:智能] OR "临床实践"[关键词:智能] OR "临床实践指南"[关键词:智能] OR "共识"[关键词:智能] OR "专家共识"[关键词:智能] OR "专业共识"[关键词:智能]) OR ( "指南"[中文标题:智能] OR "实践指南"[中文标题:智能] OR "临床指南"[中文标题:智能] OR "临床实践"[中文标题:智能] OR "临床实践指南"[中文标题:智能] OR "共识"[中文标题:智能] OR "专家共识"[中文标题:智能] OR "专业共识"[中文标题:智能])

(#1) AND (#2)

**PubMed**

#1 "guidelines as topic"[MeSH Terms]

#2 "guideline" [pt]

#3 guideline* [tiab]

#4 guidance [tiab]

#5 recommendation* [tiab]

#6 consensus [tiab]

#7 #1 OR #2 OR #3 OR #4 OR #5 OR #6

#8 massage [mesh]

#9 chiropractic [mesh]

#10 acupressure [mesh]

#11 massage [Title/Abstract]

#12 chiropractic[Title/Abstract]

#13 acupressure [Title/Abstract]

#14 "tuina"[Title/Abstract]

#15 manipulation [tiab]

#16 osteopathic [tiab]

#17 spinal [tiab]

#18 #8 OR #9 OR #10 OR #11 OR #12 OR #13 OR #14 OR #15 OR #16 OR #17

#19 “Complementary Therapies”[MeSH Terms]

#20 “Medicine, East Asian Traditional”[MeSH Terms]

#21 complementary [Title/Abstract]

#22 " East Asian Traditional"[Title/Abstract]

#23 "TCM"[Title/Abstract]

#24 "chinese medicine"[Title/Abstract]

#25 “traditional chinese”[Title/Abstract]

#26 "traditional medicine"[Title/Abstract]

#27 alternative [Title/Abstract]

#28 "oriental medicine"[Title/Abstract]

#29 "east asian medicine"[Title/Abstract]

#30 #19 OR #20 OR #21 OR #22 OR #23 OR #24 OR #25 OR #26 OR #27 OR #28 OR #29

#31 humans[mh]

#32 #7 AND #18 AND #30 AND #31

**Embase**

#1 ("guidelines as topic")/exp OR (("guideline"):it) OR ((guideline*):ab,ti) OR ((guidance):ab,ti) OR ((recommendation*):ab,ti) OR ((consensus):ab,ti)

#2 (massage)/exp OR ((chiropractic)/exp) OR ((acupressure)/exp) OR (("massage"):ab,ti) OR (("chiropractic"):ab,ti) OR (("acupressure"):ab,ti) OR (("tuina"):ab,ti) OR (("manipulation"):ab,ti) OR (("osteopathic"):ab,ti) OR (("spinal"):ab,ti)

#3 ('Complementary Therapies')/exp OR (('Medicine, East Asian Traditional')/exp) OR ((complementary):ab,ti) OR (('East Asian Traditional'):ab,ti) OR ((TCM):ab,ti) OR (('chinese medicine'):ab,ti) OR (('traditional chinese'):ab,ti) OR (("traditional medicine"):ab,ti) OR (("alternative"):ab,ti) OR (('oriental medicine'):ab,ti) OR (('east asian medicine'):ab,ti)

# 4 'human'/exp OR human

#1 AND #2 AND #3 AND #4
