# Supplementary material for: Quality appraisal of clinical practice guidelines addressing massage interventions using the AGREE II instrument
Source: Syst Rev. 2024 Mar 8;13:83. doi: 10.1186/s13643-024-02503-6 (PMC10921609; doi:10.1186/s13643-024-02503-6)
Supplement: Supplementary file 2 — Additional file 2: Appendix 2. Characteristics of guidelines concerning the contents of rigor of development. [file 13643_2024_2503_MOESM2_ESM.docx]

**Supplemental material 2** Characteristics of guidelines concerning the contents of rigor of development.

| Guideline, year | Country | Original/  Updated | Systematical search | Databases | search strategies | Study basis for massage recommendations | Methods used to determine recommendations | Peer review |
| --- | --- | --- | --- | --- | --- | --- | --- | --- |
| COSTB13, 2006 | UK | Updated | Yes | Cochrane, Medline, Health Star, Embase, Pascal, Psychoinfo, Biosis, Lilacs and IME (Indice Medico Espanol) | Yes | Systematic review, RCT, additional trials | Evidence + Expert discussion | Yes |
| SIO, 2017 | USA | Updated | Yes | Embase, MEDLINE, PsychINFO, and CINAHL. | Yes | RCT | Evidence + Expert discussion | NR |
| ACCP, 2013 | USA | Updated | Yes | Ovid MEDLINE, PubMed, and Web of Science | Yes | Systematic review; RCT | Evidence based only | Yes |
| BGS, 2013 | UK | Original | Yes | PubMed, CINAHL, AMED, PsycInfo and Scopus | Yes | NR | Evidence + Expert discussion | Yes |
| CCA, 2011 | Canada | Original | Yes | MEDLINE; EMBASE; Allied and Complementary Medicine; the Cumulative Index to Nursing and Allied Health Literature; Manual, Alternative, and Natural Therapy Index System; Alt HealthWatch; Index to Chiropractic Literature and the Cochrane Library | Yes | RCT; Systematic review | Evidence + Expert discussion | NR |
| CCGI, 2016 | Canada | Updated | Yes | Medline and Cochrane Central databases | Yes | RCT | Evidence + Expert discussion | Yes |
| ACP, 2020 | USA | Original | Yes | MEDLINE,Embase, CINAHL, PEDro, and CENTRAL | No | RCT | Evidence based only | Yes |
| OPTIMa, 2019 | USA | Original | Yes | MEDLINE, EMBASE, PsycINFO, and the Cochrane Central Register of Controlled Trials through Ovid Technologies, Inc., and CINAHL Plus through EBSCOhost, EconLit through ProQuest, Health Technology Assessment (Cochrane), and National Health Service Economic Evaluation Database (Cochrane) | NR | RCT | Evidence + Expert discussion | Yes |
| OPTIMa, 2016 | USA | Original | Yes | MEDLINE, EMBASE, PsycINFO, and the Cochrane Central Register of Controlled Trials through Ovid Technologies, Inc., and CINAHL Plus through EBSCOhost, EconLit through ProQuest, Health Technology Assessment (Cochrane), and National Health Service Economic Evaluation Database (Cochrane) | NR | Systematic review, RCT | Evidence + Expert discussion | Yes |
| CCGI, 2017 | Canada | Original | Yes | Medline and Cochrane | Yes | RCT | Evidence + Expert discussion | Yes |
| CCA, 2013 | Canada | Updated | Yes | MEDLINE, EMBASE, EMCARE, Index to Chiropractic Literature, and the Cochrane Library | Yes | RCT | Evidence + Expert discussion | NR |
| OP, 2012 | Canada | Updated | Yes | EMBASE, MEDLINE, HealthStar, PUBMED, CINAHL, PEDro Psycinfo Rehabdata, SUMsearch, Dissertatino, Abstracts International databases, and Cochrane Library. | NR | RCT | Evidence + Expert discussion | Yes |
| CCGPP, 2015 | USA | Updated | Yes | PubMed, Index to Chiropractic Literature, CINAHL, andMANTIS. | Yes | NR | Evidence + Expert discussion | NR |
| AOA, 2016 | USA | Updated | Yes | PubMed, CINAHL, Science Direct, and Springer Link databases, Cochrane Central Register of Controlled Trials (CENTRAL), MEDLINE, Embase, PEDro, OSTMED.DR, and Osteopathic Web Research, metaRegister of Controlled Trials. | Yes | NR | Evidence + Expert discussion | Yes |
| OP, 2012 | Canada | Updated | Yes | Embase, Medline, HealthStar, Pubmed, Cinahl, Pedro psycinfo Rehabdata, SUMsearch, Dissertatino, Abstracts International databases, and Cochrane Library. | NR | RCT | Evidence + Expert discussion | Yes |
| ACP, 2017 | USA | Original | Yes | MEDLINE and the Cochrane Database | Yes | RCT | Evidence based only | Yes |
| TCM Recs, 2021 | China | Original | Yes | CNKI, VIP, WanFang Data, CBM, PubMed and The Cochrane Library， | Yes | RCT | Evidence + Expert discussion | Yes |
| Chen, 2017 | China | Original | Yes | CNKI, VIP, WanFang Data, CBM, CMFD, MEDLINE | NR | RCT, non-RCT, systematic review | Evidence + Expert discussion | Yes |
| COTB, 2019 | China | Original | No | NR | NR | RCT; non-randomized controlled trial | Evidence + Expert discussion | NR |
| TCM Recs, 2022 | China | Original | Yes | CNKI, VIP, WANGFANG, CBM, PubMed, Cochrane Library | NR | RCT | Evidence + Expert discussion | Yes |
| AIMSS, 2022 | Australia | Original | No | NR | NR | NR | NR | NR |
| ASCO, 2021 | USA | Originial | Yes | PubMed, Embase, CINAHL, ISI Web of Science, and the Cochrane Central Register of Controlled Trials | NR | RCT | Evidence + Expert discussion | Yes |
| JOA, 2022 | Japan | Update | Yes | ICHU-SHI | Yes | Review and RCTs | Evidence + Expert discussion | NR |
| NICE, 2021 | UK | Updated | Yes | the OVID platform | Yes | RCT | Evidence + Expert discussion | Yes |
| NICE, 2021 | UK | Updated | Yes | the OVID platform | Yes | RCT | Evidence + Expert discussion | Yes |
| NICE, 2020 | UK | Updated | Yes | Medline, Embase, and The Cochrane Library, CINAHL, PsycINFO, AMED | Yes | Meta analysis | Evidence + Expert discussion | Yes |
| NICE, 2022 | UK | Updated | Yes | Cochrane Database of Systematic Reviews; Cochrane Central Database of Controlled Trials; International HTA database (INAHTA); MEDLINE | Yes | RCT | Evidence + Expert discussion | NR |
| NICE, 2022 | UK | Updated | Yes | Cochrane Database of Systematic Reviews; Cochrane Central Database of Controlled Trials; International HTA database (INAHTA); MEDLINE | Yes | NR | Evidence + Expert discussion | NR |
| John W. Devlin, 2018 | USA | Updated | Yes | NR | NR | RCT | Evidence + Expert discussion | NR |
| SOGC, 2017 | Canada | Updated | No | NR | NR | systematic review and meta-analysis | Conference consensus | NR |
| SD Guy, 2016 | Canada | Original | No | NR | NR | RCT | Evidence + Expert discussion | NR |
| CSMC, 2020 | China | Original | No | NR | NR | Expert consensus | Conference consensus | NR |
| CPRC, 2022 | China | Updated | Yes | NR | NR | SR, RCTs, retrospective case-control study, | Evidence + Expert discussion | Yes |
| ASCO, 2022 | America | Original | Yes | PubMed, Cochrane Library | Yes | SR, RCTs, and meta -analysis | Evidence + Expert discussion | Yes |
| Cheryl Hawk, 2021 | America | Original | Yes | PubMed | Yes | Guidelines, SR, RCTs, meta -analysis, and observational cohort study | Evidence + Expert discussion | Yes |
| Cheryl Hawk, 2020 | America | Original | Yes | PubMed, Cochrane Database of Systematic Reviews | Yes | Guidelines, SR, and meta -analysis | Evidence + Expert discussion | Yes |

COST B13: COST B13 Working Group on Guidelines for Chronic Low Back Pain; SIO: the Society for Integrative Oncology; ACCP: American College of Chest Physicians; BGS: the British Geriatrics Society; CCA: Canadian Chiropractic Association; CCGI: The Canadian Chiropractic Guideline Initiative; ACP: The American College of Physicians; OPTIMa: Ontario Protocol for Traffic Injury Management Collaboration; CCGPP: Council on Chiropractic Guidelines and Practice Parameters; AOA: The American Osteopathic Association; TCM Recs: Trustworthy Traditional Chinese Medicine Recommendations Working Group; COTB: Orthopedics and Traumatology Branch of China Association of Chinese Medicine (CACM); AIMSS: Australian Institute for Musculoskeletal Science; ASCO: American Society of Clinical Oncology; JOA: Japanese Orthopaedic Association; NICE: the National Institute for Health and Clinical Excellence; SOGC: the Society of Obstetricians and Gynaecologists of Canada Clinical Practice-Gynaecology; CSMC: Professional Committee of Spine Medicine of Chinese Association of integrated Traditional and western medicine (CAIM); CPRC, Pediatric Rehabilitation Committee of China Association Rehabilitation Medicine (CARM).
