# Supplementary material for: Quality appraisal of clinical practice guidelines addressing massage interventions using the AGREE II instrument
Source: Syst Rev. 2024 Mar 8;13:83. doi: 10.1186/s13643-024-02503-6 (PMC10921609; doi:10.1186/s13643-024-02503-6)
Supplement: Supplementary file 3 — Additional file 3: Appendix 3. The AGREE II scores of each eligible CPGs/consensus. [file 13643_2024_2503_MOESM3_ESM.docx]

| AGREE Ⅱ assessment scores of six domains of eligible consensus | | | | | | | | |
| --- | --- | --- | --- | --- | --- | --- | --- | --- |
| Domains | | Content | Median score (IQR, %) | Mean score (X±SD, %) | Score segmentation (number of included guidelines (%)) | | | |
|  |  |  |  |  | <25% | 25%~50% | 50%~75% | >75% |
| 1 | Scope and Purpose | | 26.0(21.6~44.8) | 33.2±19.2 | 3 (23.1) | 8(61.5) | 2 (15.4) | 0(0) |
| 2 | Stakeholder involvement | | 11.1 (8.9~26.0) | 18.0±15.0 | 9 (69.2) | 3 (23.1) | 1 (7.7) | 0(0) |
| 3 | Rigour of development | | 18.0 (10.0~28.9) | 19.4±15.6 | 9 (69.2) | 3 (23.1) | 1(7.7) | 0(0) |
| 4 | Clarity of presentation | | 5.6 (4.0~15.7) | 9.83±9.7 | 13（100) | 0(0) | 0(0) | 0(0) |
| 5 | Applicability | | 16.7 (10.4~25.7) | 18.0±12.6 | 9 (69.2) | 4 (30.1) | 0(0) | 0(0) |
| 6 | Editorial independence | | 0.0 (3.1~50.7) | 26.9±39.4 | 9 (69.2) | 0(0) | 0(0) | 4（30.8） |

| AGREE Ⅱ assessment scores of six domains of eligible guidelines | | | | | | | | | | | | | | | |
| --- | --- | --- | --- | --- | --- | --- | --- | --- | --- | --- | --- | --- | --- | --- | --- |
| Domains | | Content | Median score (IQR, %) | | Mean score (X±SD, %) | | Score segmentation (number of included guidelines (%)) | | | | | | | | |
|  |  |  |  |  |  |  | <25% | | 25%~50% | | 50%~75% | | >75% | | |
| 1 | Scope and Purpose | | | 75.0 (52.0~92.5) | | 73.1±21.9 | | 0 (0.0) | | 6 (16.7) | | 12 (33.3) | | 18 (50.0) |  |
| 2 | Stakeholder involvement | | | 39.0 (31.0~56.8) | | 43.7±15.0 | | 2 (5.6) | | 21 (58.3) | | 12 (33.3) | | 1 (2.8) |  |
| 3 | Rigour of development | | | 59.0 (44.3~66.5) | | 55.1±18.0 | | 3 (8.3) | | 9 (25.0) | | 20 (55.6) | | 4 (11.1) |  |
| 4 | Clarity of presentation | | | 57.5 (44.0~66.5) | | 54.7±15.9 | | 1 (2.8) | | 9 (25.0) | | 24 (66.7) | | 2 (5.6) |  |
| 5 | Applicability | | | 30.0 (17.0~48.3) | | 31.9±21.2 | | 14 (38.9) | | 14 (38.9) | | 7 (19.4) | | 1 (2.8) |  |
| 6 | Editorial independence | | | 50.0 (33.0~83.0) | | 56.1±28.2 | | 6 (16.7) | | 5 (13.9) | | 15 (41.7) | | 10 (27.8) |  |
